# Metabolic benefits afforded by estradiol and testosterone in both sexes: clinical considerations

**DOI:** 10.1172/JCI180073

**Published:** 2024-09-03

**Authors:** Franck Mauvais-Jarvis, Sarah H. Lindsey

**Affiliations:** 1 Medicine Service, Section of Endocrinology, Hormone Therapy Clinic, Southeast Louisiana VA Medical Center, New Orleans, Louisiana, USA.; 2 Deming Department of Medicine, Section of Endocrinology and Metabolism, Tulane University School of Medicine, New Orleans, Louisiana, USA.; 3 Tulane Center of Excellence in Sex-Based Biology & Medicine, New Orleans, Louisiana, USA.; 4 Department of Pharmacology, Tulane University School of Medicine, New Orleans, Louisiana, USA.

## Abstract

Testosterone (T) and 17β-estradiol (E_2_) are produced in male and female humans and are potent metabolic regulators in both sexes. When E_2_ and T production stops or decreases during aging, metabolic dysfunction develops and promotes degenerative metabolic and vascular disease. Here, we discuss the shared benefits afforded by E_2_ and T for metabolic function human females and males. In females, E_2_ is central to bone and vascular health, subcutaneous adipose tissue distribution, skeletal muscle insulin sensitivity, antiinflammatory immune function, and mitochondrial health. However, T also plays a role in female skeletal, vascular, and metabolic health. In males, T’s conversion to E_2_ is fundamental to bone and vascular health, as well as prevention of excess visceral adiposity and the promotion of insulin sensitivity via activation of the estrogen receptors. However, T and its metabolite dihydrotestosterone also prevent excess visceral adiposity and promote skeletal muscle growth and insulin sensitivity via activation of the androgen receptor. In conclusion, T and E_2_ are produced in both sexes at sex-specific concentrations and provide similar and potent metabolic benefits. Optimizing levels of both hormones may be beneficial to protect patients from cardiometabolic disease and frailty during aging, which requires further study.

## Introduction

Testosterone (T) and 17β-estradiol (E_2_) are considered male and female sex hormones, respectively, because they are secreted by gonads in the circulation at sex-specific concentrations and are involved in sexual differentiation and reproduction. E_2_, however, is not exclusively a female hormone since, for example, it is essential for erection and libido in male individuals (1). Likewise, T is not exclusively a male hormone, as it is essential for libido in female individuals (2). Most importantly, E_2_ and T are central to metabolic homeostasis of most cells and in both sexes. When E_2_ and T production stops or decreases during aging, metabolic dysfunction develops and promotes degenerative metabolic and vascular disease. Understanding the sex-specific and shared benefits of E_2_ and T in metabolic function in both sexes is critical to medicine and healthy aging. Here, we analyze sex differences and similarities in E_2_ and T benefits for metabolic homeostasis in male and female humans, including glucose and lipid metabolism, bone, vascular, adipose, muscle, and immune functions, and the prevention of metabolic dysfunction leading to cardiometabolic disease. We use the terms male and female to describe the biological sex of human subjects through the paper and we specify when animal studies are discussed. For details on mechanisms of E_2_ and T’s actions, we will refer to recent and landmark reviews.

## Origin of T and E_2_ in both sexes

**Figure 1:**
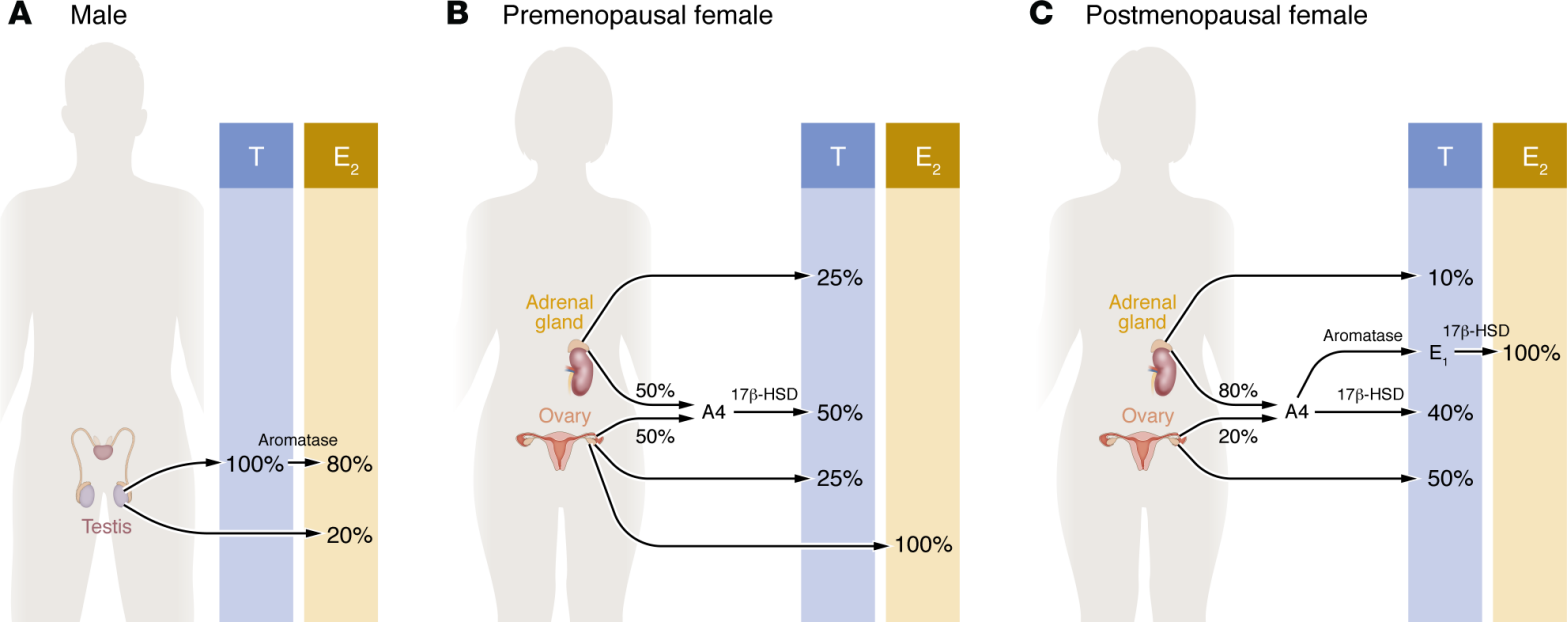
Origin of T and E_2_ in males and females. (**A**) In males, all T is produced by Leydig cells of the testis. Most E_2_ (80%) is formed via aromatization of circulating T in the periphery. The testes directly produce approximately 20% of circulating E_2_. (**B**) In females of reproductive age, the granulosa cells of the ovaries produce E2, the major circulating estrogen. T is produced by the ovary (25%), the adrenal gland (25%), and in peripheral tissues (50%) following conversion from circulating androstenedione (A4, an androgen that is equally produced by the ovary and the adrenal gland). (**C**) After menopause, estrone (E_1_) becomes the major circulating estrogen and is produced by aromatization from A4 (mainly produced by the adrenal gland) in adipose tissue. E_1_ serves as a reservoir of E_2_. T is mainly produced by the ovaries (50%) and peripheral conversion of A4 (40%). 17β-HSD, 17β-hydroxysteroid dehydrogenase.

**Figure 2:**
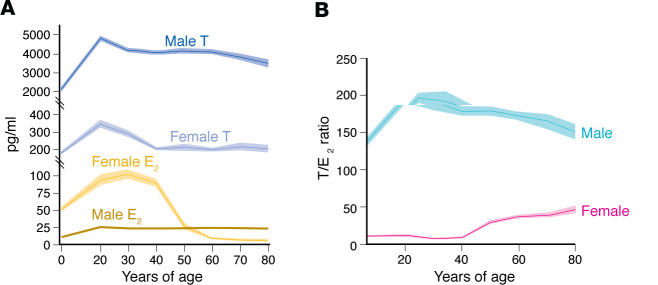
T and E_2_ concentrations in males and females. (**A**) Circulating T and E_2_ in males and females over the life span. (**B**) Ratio of T to E_2_ in males and females. Data in both panels derived from the CDC’s NHANES sex steroids data from 2013–2014 and 2015–2016 databases using sex steroids data from 2013–2014 and 2015–2016 for 7201 males and 7561 females (156, 157). In these data, total hormone (free and protein-bound) was measured using isotope dilution liquid chromatography–tandem mass spectrometry (ID-LC-MS/MS). We binned data from participants ages 6 years and up into decades and plotted as 95% confidence intervals (shown as lighter shading around averaged line). Data outside of the reported range of values were excluded (E_2_: 2.117 to 1220 pg/mL and T: 4.1 to 15,500 pg/mL).

In males, all T is produced by Leydig cells of the testis. T behaves as a hormone by binding the androgen receptor (AR), and also behaves as a prohormone that is converted in peripheral tissues to E_2_ or dihydrotestosterone (DHT), a pure AR agonist that cannot be converted to E_2_. In males, most E_2_ (80%) is formed via aromatization of circulating T in the periphery. The testes directly produce approximately 20% of circulating E_2_ (3) (Figure 1A). Circulating concentrations of E_2_ in males are half of those of females and are essential to metabolic homeostasis, as we will discuss. In females of reproductive age, the granulosa cells of the ovaries produce E_2_, the major circulating estrogen (Figure 1B). After menopause, estrone (E_1_) becomes the major circulating estrogen (4). E_1_ is produced by aromatization from the adrenal androgen androstenedione in adipose tissue (5) (Figure 1C). E_1_ is a weak estrogen and should be considered a reservoir of the more potent E_2_ in postmenopausal females. E_2_ is produced locally in extra-ovarian tissues and acts locally as a paracrine and intracrine factor (Figure 1). In females, T is the most abundant circulating active sex steroid throughout the life span (Figure 2). In females of reproductive age, T is produced by the ovary (25%), the adrenal gland (25%), and in peripheral tissues (50%), following conversion from circulating androstenedione (equally produced by the ovary and the adrenal gland) (6–9) (Figure 1B). After natural menopause, ovarian T production decreases slowly. T is mainly produced by the ovaries (50%) and via peripheral conversion from androstenedione (40%) mainly of adrenal origin (6–9). Direct adrenal production of T is minor (around 10%) (Figure 1C). Although T is ten times less abundant in the blood of females than males, in females across the life span, circulating T is 5–50 times more abundant than E_2_ (Figure 2), the implications of which we will discuss below.

## E_2_ promotes metabolic homeostasis in females

In females of reproductive age, E_2_ is instrumental to skeletal, vascular, and energy homeostasis. The central role of E_2_ in maintenance of bone metabolism, the detrimental effect of postmenopausal E_2_ deficiency on osteopenia and osteoporosis, and their prevention by estrogen therapy in postmenopausal females is evidence-based medicine (10, 11) and will not be discussed here.

### E_2_ promotes female vascular function and health.

Females with early E_2_ deficiency because of surgical oophorectomy (12, 13), premature ovarian insufficiency (14), or early menopause (15) are at increased risk of cardiovascular disease (CVD) and mortality compared with females who experience natural menopause. As we will discuss below, the vascular protection provided by E_2_ extends to males through T conversion. E_2_ protects arteries by promoting vasodilation, either through stimulation of nitric oxide (NO) production in endothelial cells or direct effects on dilatory mechanisms within vascular smooth muscle. Brachial artery flow–mediated dilation (FMD) is NO mediated (16) and is considered the gold standard for assessing macrovascular endothelial health because it is a strong predictor of future CVD (17). E_2_ increases FMD at puberty in females (18) and maintains greater FMD in reproductive-aged females versus males (19), while E_2_ deficiency after menopause reduces FMD (20). This ability of E_2_ to improve vascular tone is integral for its protection against high blood pressure, supported by the increased incidence of hypertension after surgical or early menopause (21). In rodent models that display male predominance in hypertension, ovariectomy in females increased blood pressure to the level of male rodents (22). The association of menopausal hormone therapy with hypertension is observed only with oral estrogens, especially conjugated equine estrogens (CEEs) and oral estrogen in combination with synthetic progestogens, not progesterone, highlighting the importance of differentiating endogenous versus synthetic hormones as well as route of administration (23, 24).

The second mechanism for the vascular protection provided by E_2_ relates to its ability to prevent detrimental remodeling, including fibrosis, stiffening, and calcification. Pulse wave velocity is a clinical measure of arterial stiffness and a strong predictor of cardiovascular events (25). Supporting the importance of E_2_ in protecting from arterial stiffness, females exhibited lower arterial stiffness than males only between puberty and menopause (26). Stiffness significantly increased in females at menopause (27), and in fact females developed higher arterial stiffness than age-matched males despite similar blood pressure (28). Thus, E_2_ deficiency amplifies arterial stiffness in a female-specific manner.

The third mechanism of E_2_ vascular protection involves its ability to lower atherogenic lipids (discussed in the corresponding section) and to decrease systemic inflammation. Females display a more robust immune response to infection and vaccination than males, but are more susceptible to autoimmune diseases (29). E_2_ reduces proinflammatory cytokines through direct immunomodulatory actions on immune cells (30). Atherosclerosis is a chronic inflammatory disease, characterized by elevated lipids and macrophage infiltration into the vascular wall, and mouse models show that E_2_ is atheroprotective, especially in the early stages of lesion formation (31).

**Figure 3:**
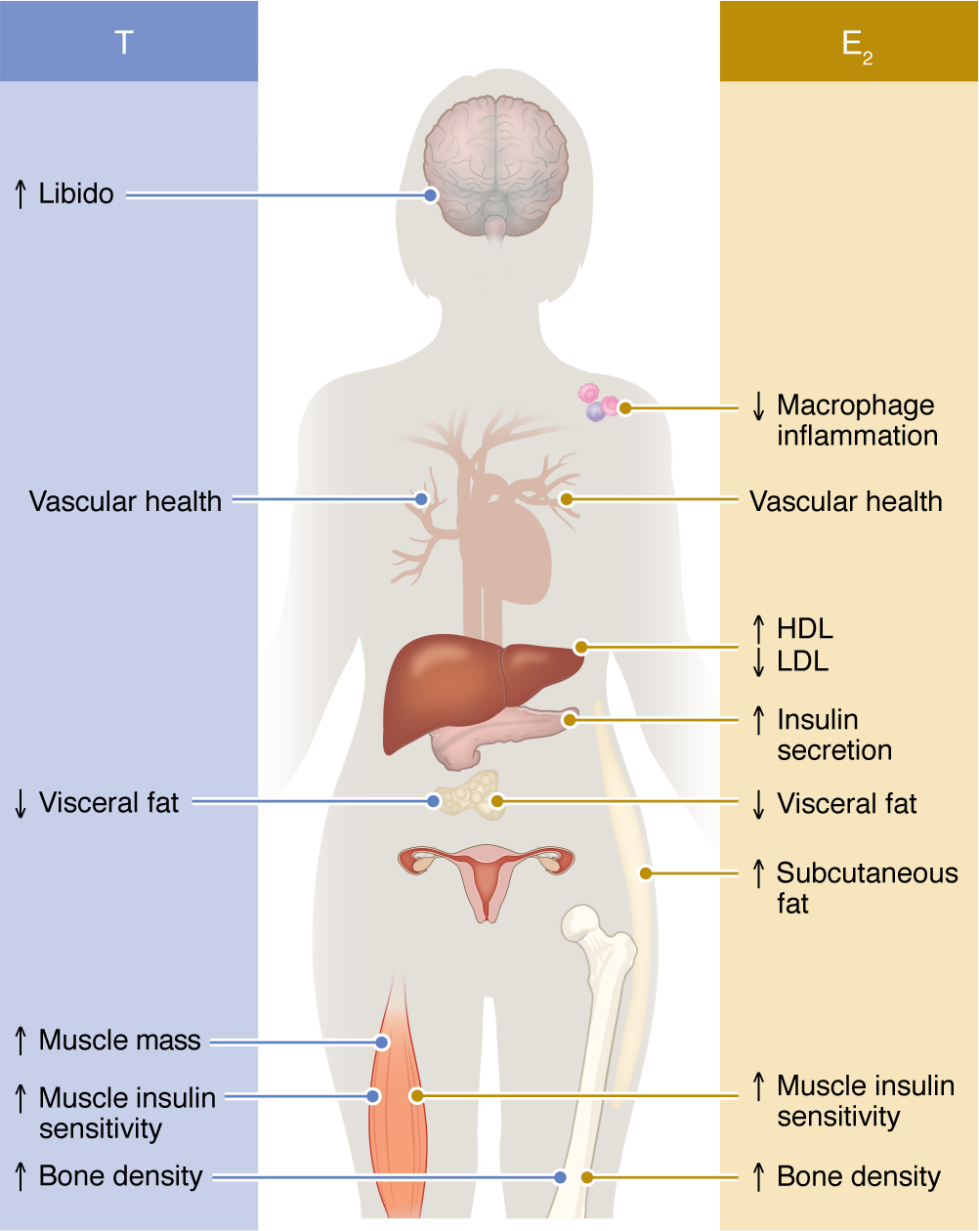
Cardiometabolic effect of E_2_ and T in females. E_2_’s effects on immune, vascular, lipid, islet, adipose, muscle, and bone biology are represented on the right, while T’s effects on vascular, adipose, muscle, and bone biology are represented on the left.

Why the vascular benefits provided by endogenous E_2_ and demonstrated in females with early E_2_ deficiency do not always translate to protection by exogenous menopausal estrogen therapy is a subject of ongoing debate. Several hypotheses have been proposed, the first of which is that endogenous E_2_ prevents or slows the progression of CVD, but does not reverse established vascular damage. If E_2_ is not restored early, then irreversible damage develops that cannot be reversed. This theory underlies the “timing hypothesis,” which postulates that E_2_ therapy started at the time of menopause in a woman with healthy arteries prevents the development of CVD, but beyond a certain point, the age- and E_2_ deficiency–related damage renders the effects of E_2_ less beneficial and potentially harmful (32). In support of this, a meta-analysis of 19 randomized controlled trials of over 40,000 postmenopausal women concluded that women who initiate estrogen therapy within 10 years of menopause show a 50% reduction in cardiovascular mortality and myocardial infarction (MI) (33). The mechanism for this early protection could be that actions mediated by ERα, but not ERβ, are protective, but prolonged E_2_ deficiency decreases the vascular ERα/ERβ ratio (34). In addition, the increased CVD observed in older postmenopausal women was related to the use of CEE therapy, not E_2_ (35). CEE contains mostly E_1_, a poor ERα agonist, along with several equine estrogens that exhibit greater affinity for ERβ (36) and are inferior to E_2_ with regard to NO production (37). Thus, CEE is likely to exhibit different vascular actions than E_2_. In summary, current evidence indicates that endogenous E_2_ prevents damage in a healthy vascular system following short-term E_2_ deficiency, but does not protect vessels exposed to prolonged E_2_ deficiency (Figure 3).

### E_2_ promotes subcutaneous lipid storage in females.

A major evolutionary function of E_2_ is to facilitate postprandial lipid storage in subcutaneous adipose tissue (SCAT) to prepare for pregnancy (38). Thus, premenopausal females carry more SCAT than males because higher circulating concentrations of E_2_ in females favors SCAT expansion and inhibits visceral adipose tissue (VAT) development. The best evidence is found in transgender individuals assigned male sex at birth who were treated with high doses of estrogens (in the presence of antiandrogens) as gender-affirming therapy. These individuals accumulated preferential SCAT in the leg and gynoid region, which increased hip circumference (39). After menopause, E_2_ deficiency leads to VAT accumulation, but it is reduced by estrogen therapy (40). As we will discuss below, T’s conversion to E_2_ is also instrumental in preventing VAT accumulation in males. In females, endogenous E_2_ also promoted lipid oxidation in skeletal muscle during fasting and exercise, but inhibited hepatic lipid oxidation during the fed and resting periods, which promoted energy storage in SCAT (38). Estrogens taken orally also increased hepatic de novo lipogenesis and triglyceride synthesis for export into very-low-density lipoproteins (VLDLs) that can be taken up by the expanded SCAT to promote lipid storage (41). After menopause, E_2_ deficiency decreases lipid oxidation and leads to disinhibition of VAT accumulation. In summary, E_2_ promotes lipid oxidation in fasting and SCAT expansion to promote lipid storage in fed and resting states while inhibiting VAT development, which produces the female gynoid phenotype.

### E_2_ promotes glucose and lipid homeostasis in females.

E_2_ is an antidiabetic hormone; thus, deficiency increases the risk of new-onset type 2 diabetes (T2D) (42). In postmenopausal women, estrogen therapy reduces the incidence of new-onset T2D and improved glycemia in women with diabetes. In postmenopausal women with T2D, estrogen therapy reduced fasting glucose and insulin as well as HbA1c (a marker of chronic hyperglycemia), and decreased the homeostatic model assessment for insulin resistance (HOMA-IR) index to a greater extent than in postmenopausal women without diabetes (40, 43). Estrogens administered orally produced a greater decrease in diabetes risk than the transdermal route (44). The stronger effect of oral estrogens on blood glucose results from first-pass liver metabolism, which better suppresses hepatic glucose output (45). The beneficial effects of endogenous E_2_ can be inferred from studies using animal models suggesting that E_2_ enhances insulin sensitivity via ERα in liver and skeletal muscle (42, 45) and protects muscle mitochondrial function, which is essential to female insulin sensitivity (46) (see also section below).

Endogenous and exogenous estrogens also protect β cell function and insulin secretion, as shown in preclinical and clinical studies (42, 47–51). This effect is less apparent clinically because the hyperbolic relationship between insulin sensitivity and β cell function (i.e., disposition index) produces a dynamic compensation of the E_2_-induced improvement in insulin sensitivity by reducing insulin secretion. Endogenous E_2_ and exogenous estrogens produce beneficial effects on cholesterol and inflammatory markers. Women experience an increase in low-density lipoprotein (LDL) cholesterol during perimenopause (52), and estrogen therapy is protective. In meta-analyses, estrogens reduce the ratio of LDL/high-density lipoprotein (HDL) cholesterol, lipoprotein (a), and the inflammatory markers E-selectin and plasminogen activator inhibitor-1 (40). In summary, E_2_ is critical in females for maintaining glucose and lipid homeostasis, which is reproduced by estrogen therapy and, as discussed below in “T promotes metabolic homeostasis in males,” is also true in males.

### E_2_ promotes mitochondrial fitness in females.

E_2_ allows women to transmit the fittest mitochondria to prevent the transmission of inherited disease (38). In female rodents, E_2_ promotes higher mitochondrial antioxidant enzyme activity, decreases reactive oxygen species production, and reduces damage to mitochondria DNA (mtDNA) compared with male rodents. In addition, E_2_ via nuclear ERα and ERβ activates a transcriptional cascade culminating in the expression of mitochondrial respiratory chain complexes (53, 54). E_2_ acting on mitochondrial ERα or ERβ also maintains mitochondrial dynamics and promotes mitochondrial fusion while attenuating fission (46, 55). In summary, E_2_ promotes female mitochondrial quality with higher respiratory capacities, biogenesis, and resistance to oxidative stress (56). Figure 3 summarizes the effects of E_2_ on female metabolic homeostasis.

## The importance of T in female metabolic homeostasis

### T production favors healthy body composition in females.

Supraphysiological levels of T in women such as those achieved during polycystic ovarian syndrome are associated with insulin resistance, visceral obesity, and T2D, demonstrating the metabolic impact of T in females (57). However, although clinical trials have focused of the effects of T supplementation in postmenopausal women with regard to libido and well being, the physiological impact of T in female metabolic homeostasis has not been explored. This lack of knowledge is surprising since, as discussed above, in females, T is always more abundant than E_2_ (Figure 2). In addition, studies have documented wide AR expression across female human tissues (58) and strong AR genomic localization in female rat tissues despite low levels of AR protein compared with male rats (59). An example that illustrates the physiological role of T in female metabolism is its conversion to active steroids in pancreatic islet β cells. Female mouse and human β cells are equipped with the enzymes aromatase and 5α-reductase (5α-R) to convert circulating T to E_2_ and DHT, respectively (60). Intracrine conversion of T to E_2_ or DHT by these enzymes was observed in female human islets, and this enhanced insulin secretion (60). In androgen-deficient women (as a result of hypopituitarism, oophorectomy, or natural menopause), T treatment that produced concentrations in the female physiological range increased lean mass (bone density and muscle mass) and decreased fat mass (61–67), improved insulin resistance (62, 68), and decreased inflammation (69, 70). T even improved aerobic capacity, muscle performance, and effort tolerance in postmenopausal females with advanced chronic heart failure (62). It is unknown to what extent the effect of T on fat mass and insulin sensitivity in females is mediated via aromatization to E_2_. However, the effect on muscle mass is likely mediated via T or DHT acting on AR, as discussed for males below. In addition, in postmenopausal females, T enhanced the effect of E_2_ in increasing bone mineral density, suggesting that T acting on AR is also important for maintenance of female bone strength (66, 67). Indeed, female mice lacking AR display reduced trabecular bone mass (71).

### Physiological T production protects female vascular health.

Hyperandrogenism in women of reproductive age has been associated with subclinical markers of atherosclerotic CVD, such as arterial stiffness, carotid intima media thickness, coronary artery calcification, endothelial dysfunction, and CVD (72). The administration of high-dose T was also associated with atherosclerosis in postmenopausal women (73). In contrast, low endogenous T in women has been prospectively associated with increased all-cause mortality and incident CVD independent of other risk factors (74). Thus, a physiological window of T seems necessary for female vascular health. Indeed, throughout the female life span, higher T concentrations within the physiological range have been associated with lower carotid intimal-medial thickness (75). Conversely, lower T concentrations were associated with carotid atherosclerosis (76, 77) and coronary artery disease (CAD) (78) in females. The mechanisms by which T promotes vascular health in females may involve a reduction in CV risk factors; apolipoprotein CIII (apoCIII) impairs the metabolism of VLDL and LDL, increasing triglycerides, and thus is a strong predictor of CAD (79). In women with surgical menopause, T added to estrogens reduced the apoCIII concentration selectively in VLDL and LDL compared with estrogens alone, which was expected to improve CAD risk (70). Addition of T to oral E_2_ counteracts the E_2_-induced rise in the inflammatory marker C-reactive protein (CRP) in postmenopausal females (69). T also promoted arterial vasodilation in postmenopausal females who were already using estrogen therapy (80), suggesting the existence of a synergism between E_2_ and T in control of blood pressure. Foam cell formation is an early event in atherosclerosis due to the uptake of LDL by macrophages in the arterial wall (81). Female mice are protected from atherosclerosis compared with males, which is believed to be due to E_2_ (31). DHT caused a dose-dependent and AR-mediated increase in macrophage cholesterol loading and atherosclerosis-related genes in cultured human male, but not female, macrophages (82, 83). T decreased atherosclerosis in female mice generated on an atherosclerosis-prone apoE-deficient background, but increased atherosclerosis in apoE-deficient male mice (84). In addition, apoE-deficient female mice lacking AR developed diet-induced obesity, dyslipidemia, and atherosclerosis (85). In summary, the physiological importance of T in female metabolic homeostasis is underestimated and may involve beneficial effects on body composition, vascular health, and prevention of atherosclerosis. Figure 3 summarizes T’s actions in female biology.

## T promotes metabolic homeostasis in males

In males, T is a hormone that binds the AR and a prohormone that provides a circulating reservoir of E_2_ and DHT. T deficiency in males leads to sexual dysfunction, depressed mood, anemia, osteoporosis, metabolic syndrome and T2D, and CVD. In the following section, we discuss the effect of T on metabolic homeostasis separated into the effects induced by E_2_ versus T/DHT.

### T-to-E_2_ conversion maintains bone mass in males.

T’s conversion to E_2_ by aromatase is instrumental to both normal bone development and preservation of healthy bone metabolism during aging. Support for the importance of E_2_ in T’s action comes from studies in young males with inactivating mutations of either ERα or aromatase who exhibit abnormal bone growth and development as well as early osteoporosis (86, 87). Furthermore, T treatment of males rendered hypogonadal using gonadotropin-releasing hormone (GnRh) agonists improved bone mineral density, but this effect was abolished with simultaneous administration of an aromatase inhibitor, which blocks T’s conversion to E_2_ (1). In aging men, E_2_ is the dominant sex steroid preventing bone resorption, whereas both E_2_ and T are important in increasing bone formation (88). It is estimated that in males, E_2_ accounts for approximately 70% of the maintenance of bone mass, with T contributing 30%.

### T is an anti-obesity hormone in males.

T deficiency promotes VAT accumulation, and the development of metabolic syndrome in males (reviewed in ref. 89).

T’s aromatization to E_2_ prevents visceral adiposity in male individuals. Orchiectomized male rodents treated with T or E_2_ remained lean, while those treated with DHT, which cannot be converted to E_2_, developed obesity (90). Similarly, in human males rendered hypogonadal using GnRh agonists, T replacement prevented VAT accumulation, an effect that was blocked in the presence of an aromatase inhibitor (1). In addition, human and rodent studies confirmed that inactivating mutations of aromatase increase VAT in males (87, 91). The mechanism by which T’s conversion to E_2_ prevents VAT in male individuals likely involves an inhibition of adipocytes and adipose progenitors as well as the promotion of lipid oxidation, as described in female individuals.

T has anti-obesity properties mediated via AR actions. In males with genetic androgen resistance (linked to CAG-repeat polymorphisms in the *AR* gene that decrease AR-mediated gene transcription), a low number of CAG repeats (which increases AR action) was associated with low adiposity and plasma insulin, demonstrating that intact AR action is necessary to prevent VAT accumulation (92). Second, male mice lacking AR developed late-onset visceral obesity and insulin resistance (93, 94). These effects of T on VAT are likely mediated via AR in skeletal muscle, as overexpression of AR selectively in muscle cells of male rats increased muscle mass, which elevated metabolic rate and reduced adipose tissue mass (95). In contrast, male adipocyte-specific AR-deficient mice exhibited no increase in VAT, demonstrating that direct AR action in adipocytes is not necessary for the control of VAT mass (96). In summary, in male individuals, T prevents VAT accumulation via E_2_’s action on ERα in muscle and adipose (like in females) as well as T/DHT’s action on AR in skeletal muscle.

### T prevents T2D in males.

Androgen deprivation therapy (ADT), the standard of treatment of prostate cancer, produces severe T deficiency and is a severe risk factor for developing T2D in males (97, 98). Moderate T deficiency also predisposes to T2D, while T replacement therapy (TRT) prevents or reverses T2D in T-deficient men (99). The antidiabetic effects of T are mediated via a decrease in VAT (described above), an increase in skeletal muscle mass and glycolytic capacity (both of which increase insulin sensitivity), and improved β cell function, as we describe below.

T improves insulin sensitivity via conversion to E_2_ and DHT, or via the effect of T itself. T promotes insulin sensitivity in skeletal muscle at least partially via an increase in peroxisome proliferator-activated receptor-γ coactivator 1-α (PGC1α), which stimulates mitochondrial biogenesis and skeletal muscle oxidative fibers, and is a molecular marker of muscle insulin sensitivity. A decrease in PGC1α in skeletal muscle was associated with insulin resistance in males (100). Similarly, men with low T exhibited low PGC1α expression in muscle (101). T’s effect on PGC1α is likely to be E_2_ mediated, as E_2_ treatment of males increases PGC1α in muscle (102). T’s improvement of insulin sensitivity also requires conversion to DHT. Dual inhibition of the T-to-DHT–converting enzymes 5α-R1 and -R2, but not inhibition of 5α-R2 alone, produced peripheral insulin resistance (103), which is associated with hepatic lipid accumulation in males (104). This suggests that T’s conversion to DHT via 5α-R1 is necessary for insulin sensitivity. T also promotes insulin sensitivity by increasing muscle mass. Surprisingly, the inhibition of T’s conversion to DHT by 5α-R inhibitors had no effect on the ability of T to increase muscle mass and strength (105), indicating that in this context, T directly binds AR and does not require conversion to DHT to promote muscle growth. T also promotes carbohydrate utilization, glycolysis, and glycogen synthesis in skeletal muscle (106, 107), which enhances insulin sensitivity via AR (106). Overexpression of AR in skeletal muscle of male mice produced hypertrophy of glycolytic muscle fibers and increased glucose metabolism (95). Activation of AR also increased glycolysis in male pancreatic islet β cells (108). In contrast, E_2_ treatment of males (which also decreases T) enhanced lipid oxidation, decreased carbohydrate oxidation during exercise (109) and in cultured male myotubes (110), and increased skeletal muscle expression of medium chain acyl-CoA dehydrogenase, a marker of lipid oxidation (102). Note that individuals assigned male sex at birth who were treated with estrogens (and androgen depletion) as gender-affirming therapy developed insulin resistance (111), suggesting that in males, E_2_ improves insulin sensitivity in the presence of intact AR action. In summary, in males, T promotes insulin sensitivity with mixed actions of E_2_ on ERα (insulin sensitivity), DHT on AR (insulin sensitivity), and T on AR (muscle mass).

T’s conversion to DHT enhances insulin secretion in male individuals. Human and rodent male β cells express 5α-R1, which is necessary to convert T to DHT and enhances glucose-stimulated insulin secretion in cultured islets (60). Male mice lacking AR in β cells (βARKO mice) developed β cell failure, leading to inadequate compensation for insulin resistance and hyperglycemia (112). βARKO islets displayed dysregulated genes involved in inflammation and insulin secretion (113). Thus, in the absence of AR in β cells, T cannot maintain normoglycemia, demonstrating the importance of the β cell AR pool to glucose homeostasis in the male. The mechanism involves DHT activation of AR, which amplifies the insulinotropic action of glucagon-like peptide 1 (GLP-1) via its receptor in human β cells, thus enhancing the hypoglycemic and anabolic actions of insulin (108, 112, 114).

T’s conversion to E_2_ is also important to β cell protection in males. First, male human β cells express aromatase, which is necessary to convert T to E_2_ and enhances insulin secretion (60). Indeed, in male mice, T’s conversion to E_2_ via aromatase was necessary to prevent β cell damage from the toxin streptozotocin (115). Second, in multiple male animal models of T2D or β cell failure, E_2_ protected male islets in vivo from diabetic injuries such as glucolipotoxicity or ER stress (47, 49, 51, 115), suggesting that T’s conversion to E_2_ is necessary to protect β cell function in males.

### Endogenous T promotes cardiovascular health in males.

Endogenous T directly protects the male cardiovascular system. T is a potent vasodilator that acutely increases coronary blood flow (116) and exerts beneficial effects on blood pressure (117). Observational studies demonstrate a direct association between low serum T concentrations and increased risk of CVD in males (118, 119). A meta-analysis of 70 studies concluded that patients with CVD exhibit lower T concentrations (120). Similarly, GnRh agonists, which suppress T production, promote vascular damage (121, 122). Accordingly, a retrospective examination of over 83,000 hypogonadal males showed that normalization of T levels by TRT decreased all-cause mortality, risk of MI, and stroke (123). Moreover, in men with T deficiency and high risk of CVD, the TRAVERSE trial using transdermal T confirmed that TRT does not increase the incidence of major adverse cardiac events (124), providing reassurance about the cardiovascular safety of TRT (125). In summary, despite controversy about T’s effects on CVD, endogenous T prevents CVD and accordingly low T predisposes to CVD. In hypogonadal men, TRT is safe regarding CVD.

Endogenous T promotes cardiovascular health in males via conversion to E_2_*,* as demonstrated by the development of endothelial dysfunction and CAD in a young male with absence of functional ERα (126, 127)**.** In middle-aged healthy males, circulating concentrations of E_2_, not T, are positively associated with FMD (128), while a reduction in plasma E_2_, through aromatase inhibition, decreases FMD (129). This effect is likely mediated via NO production, as in females. However, the beneficial effect of E_2_ in males seems to occur within a tight physiological window and in the presence of physiological T concentrations. The early Coronary Drug Project, designed to evaluate the ability of high doses of oral CEE to prevent CAD in males with prior MIs, was discontinued because of increased incidence of MI (130). Similarly, high-dose diethylstilbestrol, a synthetic estrogen, increased the incidence of atherothrombotic disease in males (131), and high-dose ethinyl estradiol, a potent synthetic estrogen used for contraception, increased CVD risk when used as a gender-affirming therapy in transgender individuals assigned male sex at birth (132). However, lower doses of CEE, ethinyl estradiol, or E_2_ for shorter duration in transgender individuals on gender-affirming therapy improved vascular function (133), enhanced endothelial function and arterial reactivity (134), and promoted endothelium-dependent vasodilation in the microcirculation (135). In older hypogonadal males, E_2_ enhanced endothelium-mediated vasorelaxation, attenuated vasoconstriction, and reduced blood pressure (136). Estradiol also induced male human coronary relaxation in vitro (137). Studies using genetically modified mice confirmed that the beneficial effects of E_2_ on vasodilation in male mice, as in female mice, are mediated by ERα (138). Taken together, these data demonstrate that E_2_ at physiological doses is beneficial for male vascular health.

The T/E_2_ ratio seems to be a critical parameter for optimal male CVD protection. In the general male population, the T/E_2_ ratio (both in pg/mL) ranges between 150 and 200 (Figure 2D). In males with existing atherosclerotic disease, a low T/E_2_ ratio (<120) was associated with increased systemic inflammation and inflammatory plaques, as well as an increased risk of future major adverse cardiovascular events compared with males with a higher T/E_2_ ratio (>160) (139). In older males, low T and high E_2_ levels (which decrease the T/E_2_ ratio) were also associated with an unhealthy artery wall on ultrasound (140, 141). In these studies, the low T/E_2_ reflected low T with higher E_2_ concentrations, but still in the physiological range. Thus, it is possible that higher E_2_ production in the face of low T reflects an endogenous compensatory increase in aromatase activity to lower E_2_ output in tissue and developing atherosclerosis. The importance of the T/E_2_ ratio and the stoichiometry of T and E_2_’s actions may explain why data in male or transgender patients receiving gender-affirming therapy with high-dose estrogens, which suppress T, display increased CVD risk (130–132). However, in transgender individuals receiving gender-affirming therapies, psychosocial stressors may also be implicated in CVD risk (142).

**Figure 4:**
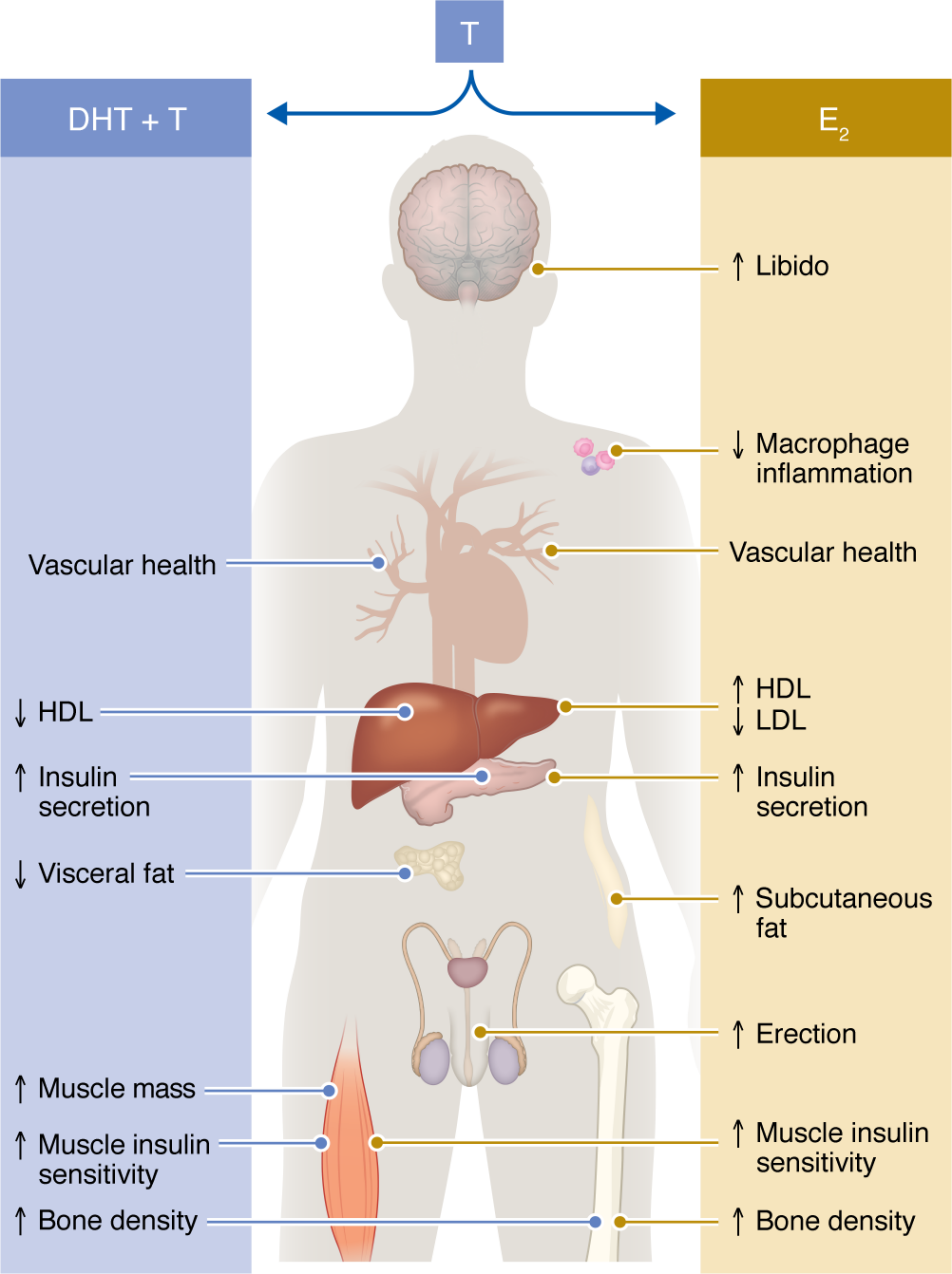
Cardiometabolic effect of T and E_2_ in males. In males, T is converted to E_2_ and DHT. T’s effects that are mediated via conversion to E_2_ on immune, vascular, lipid, islet, adipose, muscle, and bone biology as well as sexual function are represented on the right, while T’s effects mediated via direct action or conversion to DHT on vascular, lipid, islet adipose, muscle, and bone biology are represented on the left. DHT, dihydrotestosterone.

T supplementation decreases HDL in hypogonadal men (143), but produces no change in cholesterol efflux capacity (CEC) of serum HDL, a more reliable CAD risk predictor (144). This decrease in HDL is likely mediated via AR and reproduced by a selective AR modulator (145). In contrast, T is likely to improve atherogenic lipids via conversion to E_2_, as men with aromatase mutations exhibit low HDL, high LDL, and increased triglycerides, which are corrected by E_2_ treatment (87, 146). In fact, in males, oral E_2_ increased HDL (136) and decreased LDL (147), as it does in females. Oral E_2_ also decreased triglyceride and homocysteine (147). In summary, in males, T promotes vascular protection via conversion to E_2,_ likely by increasing NO and promoting a less atherogenic lipid profile. Consequently, low T, which is associated with low E_2_, predisposes to CVD. Figure 4 summarizes T’s actions in males.

## Conclusions and clinical implications

T and E_2_ are produced in both sexes at sex-specific concentrations and share similar and potent metabolic functions. The loss of E_2_ after menopause in females and the decrease in T in aging males both produce metabolic dysfunction and are serious health threats leading to cardiometabolic disease and frailty. The reason that these important metabolic mediators are not prescribed more often relates to myths about the danger of hormones. In particular, there are persistent misconceptions about the risks of estrogen-based therapies in females (148–154). Apart from the purported risk of breast cancer, which has been attributed to synthetic progestins, confusion about the risks of estrogens lies in the too often ignored biological difference between synthetic hormones like CEE, which is associated with CVD, and endogenous and bioidentical E_2_, which is not associated with negative CVD outcomes. In the case of males and T, myths about risk of prostate cancer and CVD along with its cultural associations with illegally enhancing athletic performance and toxic masculinity has created resistance to consider aging as a treatable condition of T deficiency (155).

It is not known what the role of T in female metabolism is. Is it mediated via T or DHT acting on AR, as animal studies suggest, or is T an additional reservoir for local E_2_ synthesis in tissues? Clinical trials assessing the effect of T supplementation in postmenopausal women to achieve serum concentrations in the upper limit of female physiology should be considered to ascertain its ability to improve muscle and metabolic function along with its beneficial effects on libido.

Anecdotally, male patients on TRT often enquire about their E_2_ levels due to fear of “too much female hormone.” Mens’ health clinics even prescribe aromatase inhibitors to suppress E_2_ production while raising T concentrations. However, we discussed the essential role of T’s conversion to E_2_ in male bone and vascular health, as well as glucose and lipid homeostasis (not to mention libido and erectile function). Thus, it is our view that E_2_ should not be suppressed in men, and in fact clinical trials of E_2_ supplementation should be considered in some men on TRT to decrease LDL cholesterol and improve endothelial function.

Finally, current laboratory measurements of serum T and E_2_ levels (total or free) poorly reflect tissue and cellular T and E_2_ concentrations, catabolism, and elimination. Novel assays that provide accurate measures of cellular T and E_2_ outputs will be informative in clinical studies and are desperately needed.

## Version 1 09/03/2024

Electronic publication
